# Re-authoring the altered self: the impact of a three-phase narrative nursing intervention on body image and distress in oral squamous cell carcinoma

**DOI:** 10.3389/fpsyg.2026.1819611

**Published:** 2026-05-12

**Authors:** Na Tian, Qing Liu, Huijie Wang, Qigen Fang

**Affiliations:** 1 The Affiliated Cancer Hospital of Zhengzhou University & Henan Cancer Hospital, Zhengzhou, China; 2 Department of Head Neck, The Affiliated Cancer Hospital of Zhengzhou University & Henan Cancer Hospital, Zhengzhou, China

**Keywords:** body image disturbance, narrative nursing intervention, neoadjuvant immunochemotherapy, oral squamous cell carcinoma, psychological distress

## Abstract

**Background:**

Locally advanced oral squamous cell carcinoma (OSCC) and its surgical treatment precipitate profound body image disturbance and psychological distress. With the increasing adoption of neoadjuvant immunochemotherapy (NICT), patients now experience fundamentally different treatment trajectories, yet the psychological implications of this paradigm shift remain poorly understood.

**Methods:**

This prospective, comparative mixed-methods cohort study investigated longitudinal trajectories of body image disturbance (Body Image Scale, BIS) and psychological distress (Hospital Anxiety and Depression Scale, HADS) in OSCC patients undergoing either NICT followed by surgery (*n* = 77) or upfront surgery (US; *n* = 77) following propensity score matching. Assessments occurred at baseline, post-neoadjuvant therapy (for the NICT group) or immediately pre-surgery (for the US group), at discharge, and 3 months post-surgery. Linear mixed-effects models analyzed time-by-group interactions. A qualitative phenomenological phase (*n* = 15) explored subjective experiences.

**Results:**

Significant time-by-group interactions were observed for both BIS [*F*(3,456) = 18.5, *p* < 0.001] and HADS-Total (*F*(3,456) = 14.2, *p* < 0.001). The NICT group exhibited elevated distress pre-surgery (T1: HADS-Total 15.8 vs. 12.9, *p* = 0.004) but demonstrated superior recovery by three months (T3: BIS 11.5 vs. 14.2, *p* = 0.002; HADS-Total 12.2 vs. 15.5, *p* < 0.001). Qualitative analysis revealed divergent psychological experiences: NICT patients endured prolonged anticipatory grief (“saying goodbye slowly”), while US patients experienced traumatic shock (“waking up to a stranger”).

**Conclusion:**

NICT and US pathways generate fundamentally different psychological trajectories. Despite heightened pre-surgical distress, NICT patients demonstrate more robust psychological recovery, potentially facilitated by gradual adaptation. These findings support pathway-specific psychosocial interventions.

## Introduction

Locally advanced oral squamous cell carcinoma (OSCC) poses a profound clinical challenge, not only due to its tumor biology but also because of the severe aesthetic and functional morbidity associated with its treatment (Jagadeesan et al., 2024). For patients scheduled for major reconstructive surgery, the psychological toll is immense (Jehn et al., 2019). The radical alteration of facial features fundamentally disrupts a patient’s self-identity, frequently precipitating severe body image disturbance and psychological distress (Wang et al., 2024).

Historically, the standard of care for these patients has been primary surgical resection and reconstruction without prior systemic therapy, categorized as upfront surgery (US) (Kim et al., 2026). However, the therapeutic paradigm is rapidly evolving. Neoadjuvant immunochemotherapy (NICT), particularly regimens utilizing PD-1 inhibitors combined with platinum-based chemotherapy, is increasingly administered prior to planned ablative surgery (Wu et al., 2026; Zhou et al., 2025; Fang et al., 2025). While the oncological efficacy and survival benefits of NICT are the subjects of rigorous ongoing investigation, the distinct psychological implications of this altered treatment timeline remain poorly understood (Wei et al., 2025).

The divergence in these clinical pathways creates fundamentally different psychological environments. Patients undergoing US face an immediate and violent confrontation with a surgically altered body, experiencing a sudden loss of bodily integrity without adequate time for psychological preparation (Aminnudin et al., 2020). Conversely, the NICT pathway introduces a prolonged pre-surgical phase characterized by a profound psychological duality. As the visible tumor shrinks, patients often experience a complex expectation shift and the burden of “false hope.” They are forced to manage the systemic side effects of treatment, such as fatigue and rash, while simultaneously navigating the anticipatory anxiety and gradual grief associated with their impending, inevitable surgical disfigurement (Pompili et al., 2026; Christodoulis et al., 2026). Despite these stark phenomenological differences, current psychosocial care models rarely differentiate between the unique psychological trajectories of these two cohorts. There is a critical need to map how body image and psychological distress fluctuate over time depending on the treatment pathway to inform optimally timed support strategies.

Therefore, this prospective, comparative, mixed-methods observational cohort study was designed to investigate the distinct psychological experiences of OSCC patients undergoing these differing treatment pathways. The primary objective of the quantitative phase is to evaluate and compare the longitudinal trajectories of body image disturbance and psychological distress between the NICT and US cohorts. Subsequently, the qualitative phenomenological phase aims to deeply explore the subjective experiences of these patients, exploring the nuanced ways they perceive their altered bodies and respond to a targeted “Three-Phase Narrative Reconstruction” nursing intervention.

## Methods

### Ethical considerations

This study was conducted in strict adherence to the ethical principles outlined in the Declaration of Helsinki. The research protocol received formal ethical approval from the Institutional Review Board of Henan Cancer Hospital.

### Study design

A prospective, comparative, mixed-methods observational cohort was designed to investigate the psychological experiences of patients undergoing different treatment pathways for their condition. The research was structured in two distinct phases: first, a quantitative longitudinal component that evaluated and compared the trajectories of body image disturbance and psychological distress between two clinical cohorts—those NICT followed by surgery versus those undergoing US. This quantitative phase was designed to capture the temporal patterns and statistical differences in psychological outcomes between the two groups over the course of their treatment. Subsequently, a qualitative phenomenological phase was conducted to delve deeper into the subjective experiences of participants, exploring the nuanced ways in which patients from each clinical pathway perceived and responded to their altered body image and psychological challenges, as well as their engagement with narrative-based nursing interventions (Supplementary Figure S1).

The reporting of the quantitative observational component adheres to the Strengthening the Reporting of Observational Studies in Epidemiology guidelines. The qualitative phenomenological component was conducted and reported in accordance with the Consolidated criteria for Reporting Qualitative research checklist.

### Patient enrollment

The study was conducted at a tertiary cancer center (Henan Cancer Hospital) between January 2020 and December 2024. Cohort allocation was non-randomized and determined by the institutional Multi-Disciplinary Team based on current official guidelines and tumor resectability, resulting in two groups: NICT Cohort comprised patients receiving 2 or more cycles of NICT (e.g., PD-1 inhibitors combined with platinum-based chemotherapy) prior to planned ablative and reconstructive surgery, while Group US consisted of patients proceeding directly to primary surgical resection and reconstruction without prior systemic therapy. Eligibility criteria included: (1) patients aged 18–75 years; (2) a histopathologically confirmed diagnosis of locally advanced OSCC scheduled for major reconstructive surgery (free flap or pedicle flap); and (3) the ability to communicate in Chinese. Exclusion criteria were: (1) a previous history of head and neck radiation or surgery; (2) a severe pre-existing psychiatric illness; or (3) the presence of distant metastasis.

### The intervention protocol

The nursing intervention was based on a core “Three-Phase Narrative Reconstruction” protocol, which was delivered to both cohorts. However, the timing and specific focus of each phase were carefully adapted to align with the patients’ distinct clinical pathways. Intervention fidelity was ensured through weekly supervision sessions and audio-recording of 10% of sessions for independent review.

Phase I served as the Pre-operative Proactive Intervention. For patients in NICT, narrative sessions were initiated during the infusion cycles. In this context, nurses focused on helping patients manage the psychological duality inherent to NICT. This involved addressing the burden of systemic side effects, such as fatigue and rash, while simultaneously guiding them through the complex expectation shifts and the phenomenon of “false hope” that can arise as the visible tumor shrinks prior to the planned surgery. For Group US, sessions were concentrated in the immediate days prior to the operation. Here, the focus was on rapid psychological preparation through Visual and Cognitive Rehearsal, utilizing 3D-anatomical models to help patients mentally prepare for the abrupt and significant facial alteration resulting from the surgery.

Phase II, termed Post-operative Progressive Desensitization, was standardized and implemented for both groups from Day +3 to Day +14. This phase incorporated tactile grounding techniques and a specialized “Buffer” Mirror Therapy. During this process, the nurse’s guided narrative was used to help patients reframe the initial shock of seeing their reconstructed site, fostering a more gradual and supported adjustment.

Finally, Phase III, known as Narrative Re-authoring, took place from Day +14 through Month 3. This phase involved bi-weekly deconstruction sessions where patients could explore and make sense of their experiences. A key component was the maintenance of a “Survival Journal,” which served as a tool for patients to document “Unique Outcomes”—moments of strength or progress that contradicted the dominant narrative of illness. This practice was designed to help patients construct a new, post-illness identity and facilitate successful social reintegration.

### Data collection

Longitudinal quantitative data were collected at four time points to map the trajectory of psychosocial outcomes. Baseline assessments (T0) were conducted post-MDT decision. Subsequent data were gathered post-neoadjuvant therapy or immediately pre-surgery (T1), at hospital discharge (T2; ~2–3 weeks post-surgery), and at 3 months post-surgery (T3). At these intervals, participants completed the Body Image Scale (BIS) to assess body image disturbance and the Hospital Anxiety and Depression Scale (HADS) to measure psychological distress.

To complement these quantitative data, a qualitative exploratory component was incorporated. At the three-month follow-up (T3), semi-structured interviews were conducted with a purposive sample of 6–8 participants from each group, a sample size guided by the principle of data saturation, which is standard for phenomenological inquiry.

### Sample size

The sample size was determined based on the primary quantitative outcome of the BIS score at the three-month follow-up (T3). To detect a clinically meaningful medium effect size (Cohen’s d = 0.5) in BIS scores between the NICT and US groups, with a two-tailed significance level (*α*) of 0.05 and a desired power (1 − *β*) of 0.80, a power analysis indicated a requirement of 64 participants per group. To account for an anticipated attrition rate of approximately 20% due to the longitudinal nature of the study and the potential for clinical complications, the target enrollment was set at a minimum of 77 participants per cohort, for a total sample of 154 patients.

### Data analysis

Quantitative data analysis was performed using SPSS Version 28.0. To mitigate selection bias and account for differences in baseline covariates between the NICT and US groups, Propensity Score Matching (PSM) was initially employed. Following the matching procedure, the balance of baseline demographic and clinical characteristics between the two groups was assessed using independent t-tests for continuous variables and Chi-square tests for categorical variables. To evaluate the primary outcomes, a Linear Mixed-Effects Model (LMM) was utilized. This approach was selected to analyze the main effects of time, group assignment (NICT vs. US), and the time-by-group interaction on BIS and HADS scores. For all statistical tests, a two-tailed *p*-value of less than 0.05 was established as the threshold for statistical significance.

Qualitative data analysis was guided by the principles of Reflexive Thematic Analysis (RTA) as outlined by Braun and Clarke. Transcripts from semi-structured interviews were systematically coded and thematically organized to capture the nuanced psychological experiences of participants. The analytical process was specifically attuned to identifying divergent themes between the two groups. A key area of exploration was whether the NICT group, which endured a prolonged pre-surgical phase characterized by living with a cancer diagnosis, experienced a distinct psychological or “grief trajectory” compared to the more immediate and abrupt alteration in body integrity faced by the US.

## Results

### Baseline data

**Figure 1:**
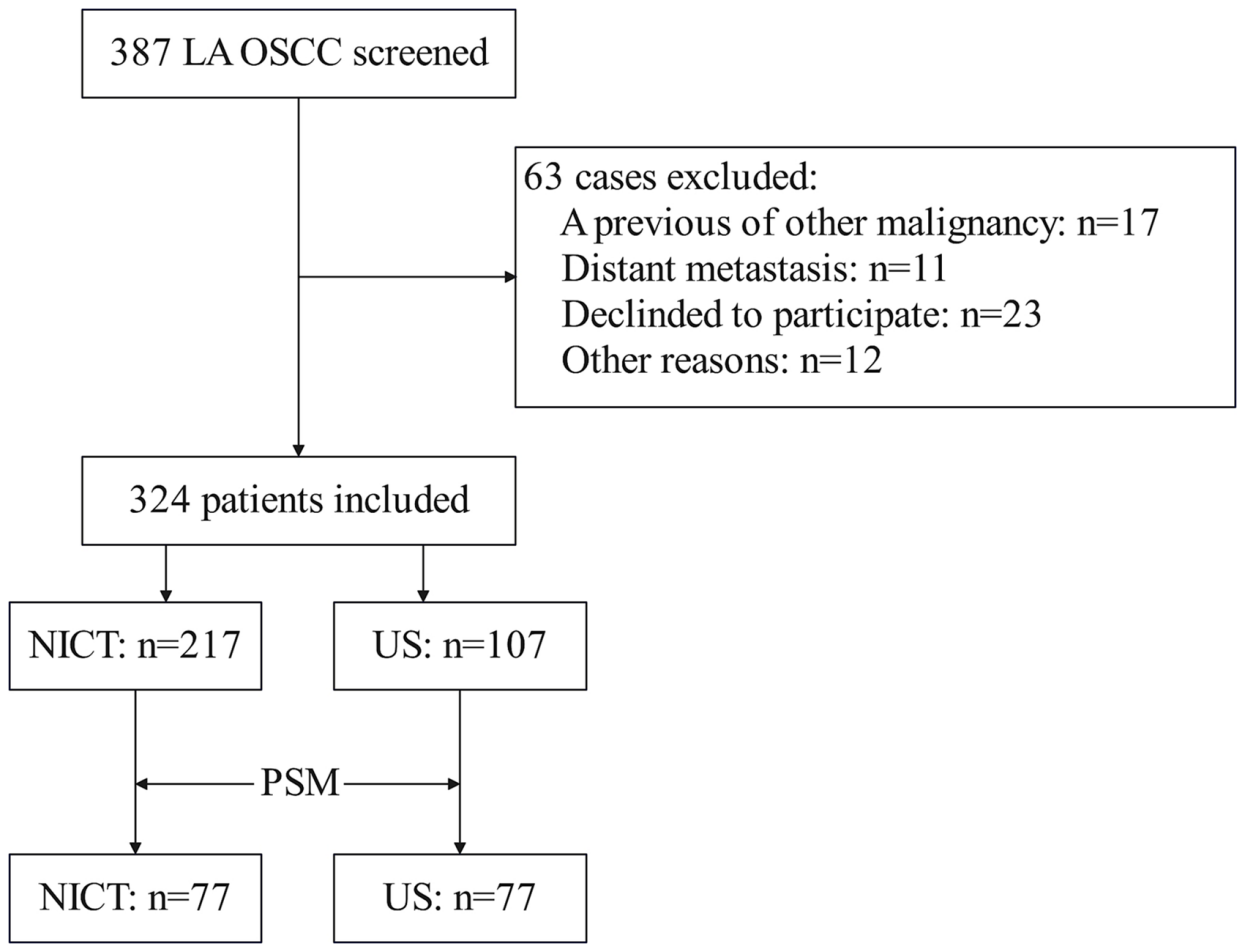
CONSORT flow diagram of participant recruitment, allocation, and matching.

A total of 387 patients with locally advanced OSCC were initially reviewed, and 63 patients were excluded. The remaining 324 patients were allocated to either NICT cohort (*n* = 217) or US cohort (*n* = 107) (Figure 1).

Prior to matching, the US group was slightly older (mean age 58.2 vs. 55.1 years, *p* = 0.041) and had a lower proportion of node-positive disease (cN+: 54.2% vs. 68.2%, *p* = 0.013) compared to the NICT group. Other covariates, including sex, cT stage, smoking status, alcohol consumption, tumor differentiation, and CPS, were similarly distributed between the groups (*p* > 0.05 for all). After PSM, all covariates were well balanced, with no statistically significant differences between the NICT and US groups (*p* > 0.05 for all comparisons) (Table 1).

**Table 1:** Baseline demographic and clinical characteristics of patients before and after propensity score matching (PSM).

Characteristic	Before PSM			After PSM		
	NICT (*n* = 217)	US (*n* = 107)	*p*-value	NICT (*n* = 77)	US (*n* = 77)	*p*-value
Age (years), mean ± SD	55.1 ± 9.8	58.2 ± 10.3	0.041	56.8 ± 9.5	57.1 ± 9.9	0.762
Sex, *n* (%)			0.327			0.854
Male	142 (65.4)	75 (70.1)		52 (67.5)	53 (68.8)	
Female	75 (34.6)	32 (29.9)		25 (32.5)	24 (31.2)	
cT stage, *n* (%)			0.184			0.921
T2	48 (22.1)	30 (28.0)		20 (26.0)	21 (27.3)	
T3	86 (39.6)	43 (40.2)		32 (41.6)	30 (39.0)	
T4	83 (38.2)	34 (31.8)		25 (32.5)	26 (33.8)	
cN stage, *n* (%)			0.013			0.855
N0	69 (31.8)	49 (45.8)		28 (36.4)	29 (37.7)	
N+	148 (68.2)	58 (54.2)		49 (63.6)	48 (62.3)	
Smoking status, *n* (%)			0.467			0.738
Never	93 (42.9)	42 (39.3)		33 (42.9)	35 (45.5)	
Current/former	124 (57.1)	65 (60.7)		44 (57.1)	42 (54.5)	
Alcohol consumption, *n* (%)			0.604			0.868
Never	101 (46.5)	52 (48.6)		37 (48.1)	38 (49.4)	
Current/former	116 (53.5)	55 (51.4)		40 (51.9)	39 (50.6)	
Differentiation, *n* (%)			0.714			0.638
Well	54 (24.9)	24 (22.4)		18 (23.4)	19 (24.7)	
Moderate	98 (45.2)	52 (48.6)		36 (46.8)	37 (48.1)	
Poor	65 (30.0)	31 (29.0)		23 (29.9)	21 (27.3)	
CPS, median (IQR)	10 (5–20)	10 (5–20)	0.839	10 (5–20)	10 (5–20)	0.912
Primary defect, *n* (%)			0.211			0.891
Soft tissue only	89 (41.0)	51 (47.7)		35 (45.5)	36 (46.8)	
Composite	128 (59.0)	56 (52.3)		42 (54.5)	41 (53.2)	
Reconstruction, *n* (%)			<0.001			0.752
Pedicled flap	119 (54.8)	34 (31.8)		30 (39.0)	32 (41.6)	
Free flap	98 (45.2)	73 (68.2)		47 (61.0)	45 (58.4)	

PSM, propensity score matching; NICT, neoadjuvant immunochemotherapy; US, upfront surgery; SD, standard deviation; CPS, combined positive score; IQR, interquartile range.

*p*-values for continuous variables were calculated using independent *t*-tests (age) or Mann–Whitney U tests (CPS); categorical variables were compared using chi-square tests. Statistical significance was set at *p* < 0.05 (two-tailed). The *p*-values after PSM confirm successful balance between the matched groups.

### Quantitative outcomes

**Figure 2:**
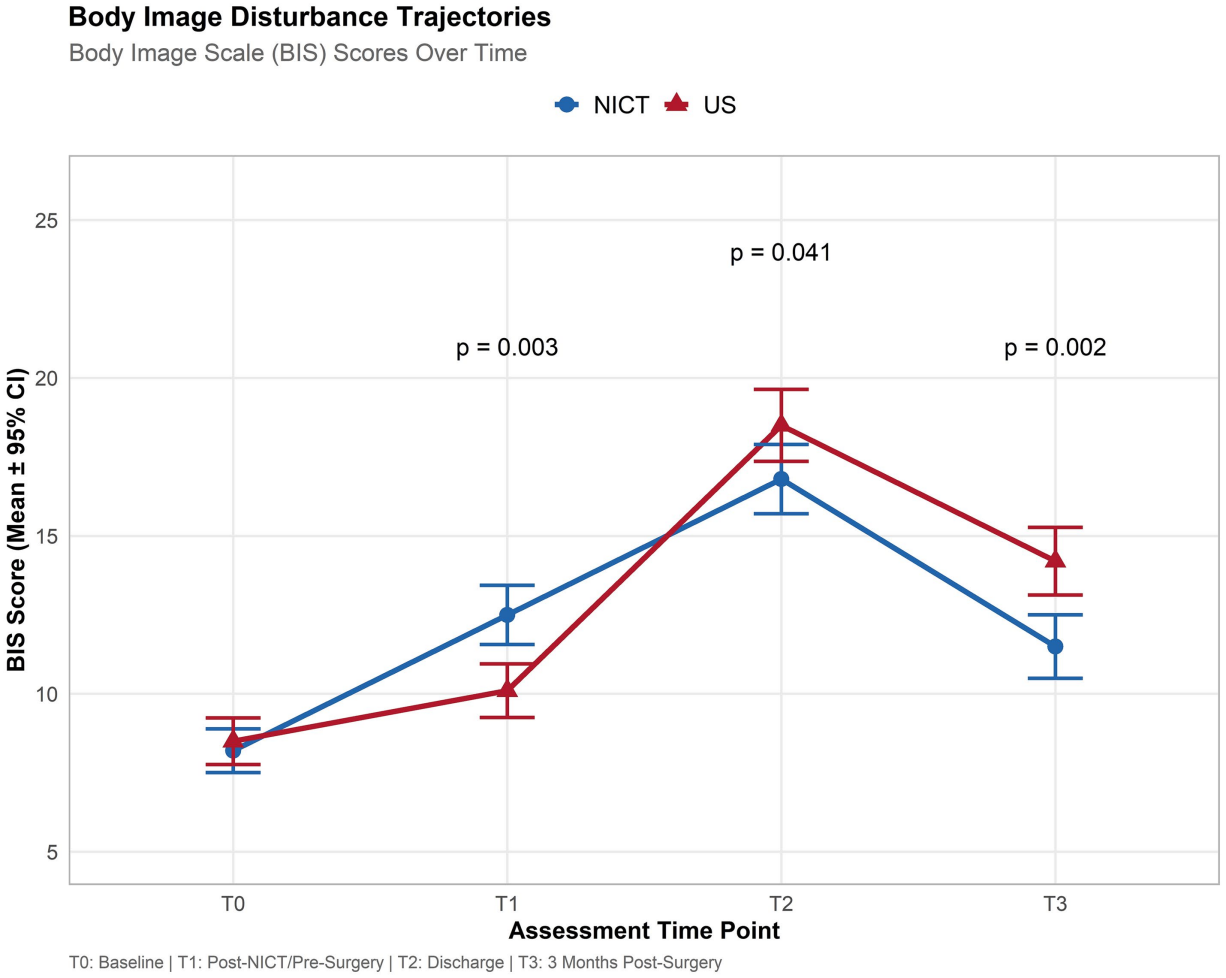
Longitudinal trajectories of body image disturbance in patients with oral squamous cell carcinoma undergoing neoadjuvant immunochemotherapy versus upfront surgery. Time point (*x*-axis) and BIS Score (*y*-axis).

In BIS assessment, at baseline (T0), both groups reported comparable levels of body image disturbance, with mean scores of 8.2 (SD = 3.1) in the NICT group and 8.5 (SD = 3.3) in the US group (*p* = 0.562), confirming successful matching after propensity score matching. At T1, which corresponded to the post-neoadjuvant phase for the NICT group and the immediate pre-surgical period for the US group, a significant divergence emerged. The NICT group experienced a marked increase in body image disturbance, with a mean score of 12.5 (SD = 4.2), while the US group showed only a modest elevation to 10.1 (SD = 3.8). This between-group difference was statistically significant (*p* = 0.003). At hospital discharge (T2), both groups reached their peak levels of body image disturbance. Interestingly, the US group reported a slightly higher mean score of 18.5 (SD = 5.1) compared to 16.8 (SD = 4.9) in the NICT group, a difference that reached statistical significance (*p* = 0.041). By the three-month follow-up (T3), a striking reversal in trajectories was observed. The NICT group demonstrated a rapid and substantial improvement, with mean BIS scores declining to 11.5 (SD = 4.5). In contrast, the US group showed more limited improvement, with scores remaining elevated at 14.2 (SD = 4.8). This between-group difference was highly significant (*p* = 0.002) (Table 2 and Figure 2).

**Table 2:** Descriptive statistics for body image scale (BIS) scores over time with between-group comparisons.

Time point	Description	NICT group mean (SD)	US group mean (SD)	*p*-value^*^
T0	Baseline (post-MDT)	8.2 (3.1)	8.5 (3.3)	0.562
T1	Post-NICT/pre-surgery	12.5 (4.2)	10.1 (3.8)	**0.003**
T2	Hospital discharge	16.8 (4.9)	18.5 (5.1)	**0.041**
T3	3 months post-surgery	11.5 (4.5)	14.2 (4.8)	**0.002**

*p-values are derived from *post-hoc* pairwise comparisons following the Linear Mixed-Effects Model, with Bonferroni adjustment for multiple comparisons. Bold values indicate statistical significance was set at *p* < 0.05.

Further LMM results provided statistical confirmation of these observed patterns. There was a highly significant main effect of Time [*F*(3, 456) = 145.2, *p* < 0.001], confirming that body image disturbance fluctuated significantly throughout the treatment journey across both groups. The main effect of Group was not significant [*F*(1, 152) = 0.9, *p* = 0.342], indicating that when averaging across all time points, the overall levels of disturbance were similar between the two cohorts. However, the critical Time-by-Group interaction was highly significant [*F*(3, 456) = 18.5, *p* < 0.001], demonstrating that the trajectories of body image disturbance over time were fundamentally different between the NICT and US groups (Table 3).

**Table 3:** Linear mixed-effects model results for BIS scores.

Effect	*F*-statistic	df	*p*-value
Main effect of time	*F*(3, 456) = 145.2	< 0.001	**< 0.001**
Main effect of group	*F*(1, 152) = 0.9	0.342	0.342
Time-by-group interaction	*F*(3, 456) = 18.5	**< 0.001**	**< 0.001**

Bold values indicate statistical significance was set at *p* < 0.05.

**Figure 3:**
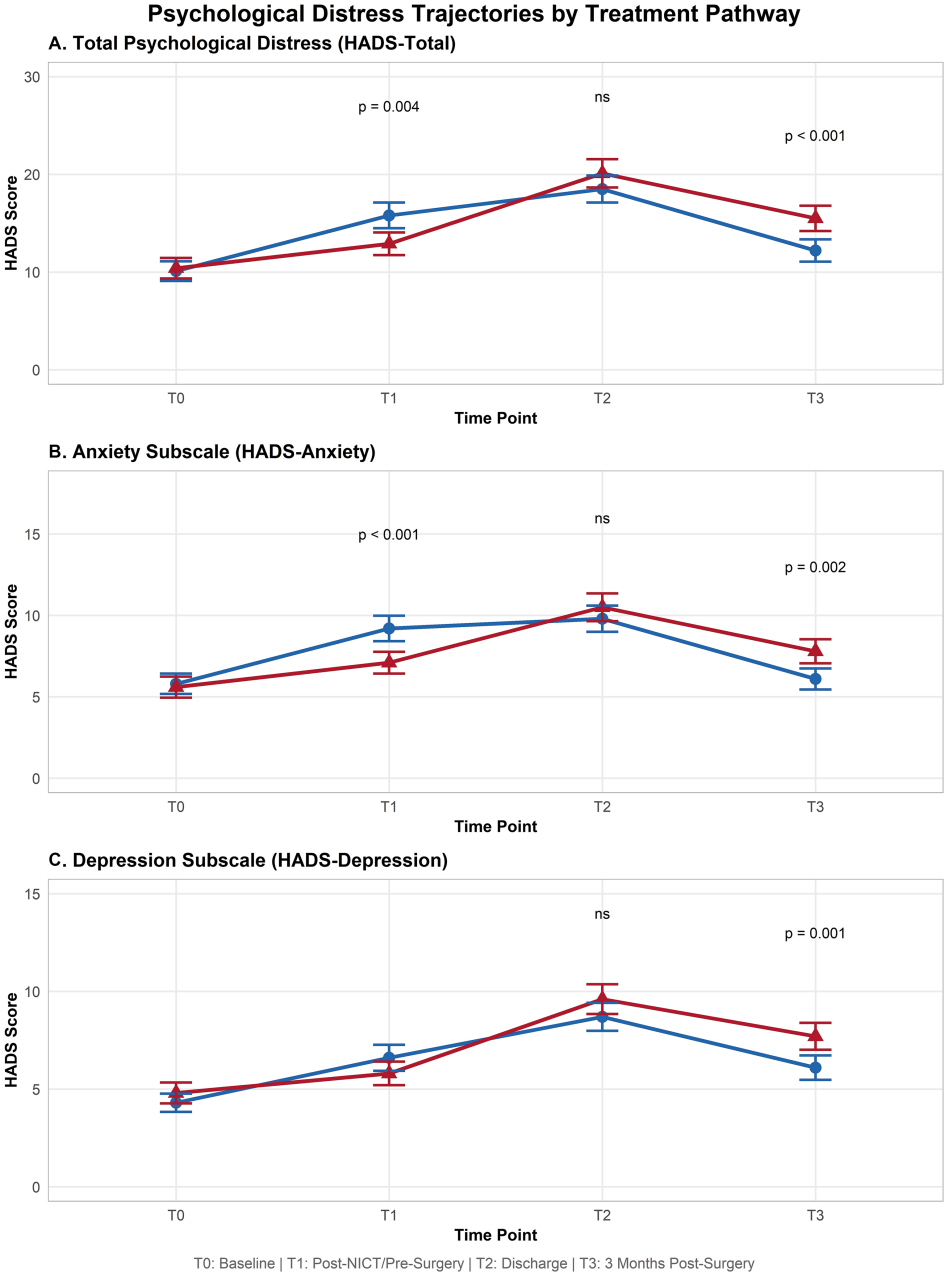
Differential trajectories of psychological distress across treatment pathways. Time point (*x*-axis) and HADS Score (*y*-axis). **(A)** for HADS-Total; **(B)** for HADS-Anxiety; **(C)** for HADS-Depression.

In HADS analysis, at baseline (T0), HADS-Total scores were comparable between the NICT group (mean = 10.1, SD = 4.5) and the US group (mean = 10.4, SD = 4.7; *p* = 0.712). By T1, a significant divergence emerged, with the NICT group reporting substantially higher total distress (mean = 15.8, SD = 5.9) compared to the US group (mean = 12.9, SD = 5.2; *p* = 0.004). At T2 (hospital discharge), both groups reached their peak distress levels, with the US group demonstrating a slightly higher mean score (20.1, SD = 6.5) compared to the NICT group (18.5, SD = 6.2), although this difference did not reach statistical significance (*p* = 0.124). By T3, however, a significant between-group difference emerged once again. The NICT group showed robust improvement, with scores declining to 12.2 (SD = 5.1), while the US group remained more distressed, with a mean score of 15.5 (SD = 5.8; *p* < 0.001) (Table 4 and Figure 3).

**Table 4:** Descriptive statistics for HADS scores over time with between-group comparisons.

Time point	Scale	NICT group mean (SD)	US group mean (SD)	*p*-value^*^
T0	HADS-total	10.1 (4.5)	10.4 (4.7)	0.712
	HADS-anxiety	5.8 (2.8)	5.6 (2.9)	0.689
	HADS-depression	4.3 (2.1)	4.8 (2.4)	0.198
T1	HADS-total	15.8 (5.9)	12.9 (5.2)	**0.004**
	HADS-anxiety	9.2 (3.5)	7.1 (3.0)	**< 0.001**
	HADS-depression	6.6 (3.0)	5.8 (2.7)	0.092
T2	HADS-total	18.5 (6.2)	20.1 (6.5)	0.124
	HADS-anxiety	9.8 (3.6)	10.5 (3.8)	0.258
	HADS-depression	8.7 (3.2)	9.6 (3.4)	0.105
T3	HADS-total	12.2 (5.1)	15.5 (5.8)	**< 0.001**
	HADS-anxiety	6.1 (2.9)	7.8 (3.3)	**0.002**
	HADS-depression	6.1 (2.8)	7.7 (3.1)	**0.001**

*p-values are derived from *post-hoc* pairwise comparisons following the linear mixed-effects model, with Bonferroni adjustment for multiple comparisons. Bold values indicate statistical significance was set at *p* < 0.05.

The LMM for HADS-Total revealed a significant main effect of Time [*F*(3, 456) = 121.5, *p* < 0.001], confirming that psychological distress fluctuated significantly across the treatment trajectory. The main effect of Group was not significant [*F*(1, 152) = 2.1, *p* = 0.149], indicating comparable overall distress levels when averaged across all time points. Crucially, the Time-by-Group interaction was highly significant [*F*(3, 456) = 14.2, *p* < 0.001], confirming that the pattern of psychological distress over time differed fundamentally between the two cohorts (Table 5).

**Table 5:** Linear mixed-effects model results for HADS scores.

Outcome	Effect	*F*-statistic	df	*p*-value
HADS-total	Main effect of time	*F*(3, 456) = 121.5	< 0.001	**< 0.001**
	Main effect of group	*F*(1, 152) = 2.1	0.149	0.149
	Time-by-group interaction	*F*(3, 456) = 14.2	**< 0.001**	**< 0.001**
HADS-anxiety	Time-by-group interaction	*F*(3, 456) = 10.8	**< 0.001**	**< 0.001**
HADS-depression	Time-by-group interaction	*F*(3, 456) = 6.9	**< 0.001**	**< 0.001**

Bold values indicate statistical significance was set at *p* < 0.05.

Examination of the anxiety subscale provided further insight into the differential psychological experiences of the two groups. At baseline, anxiety levels were nearly identical (NICT: 5.8, SD = 2.8; US: 5.6, SD = 2.9; *p* = 0.689). At T1, however, the NICT group exhibited markedly elevated anxiety (mean = 9.2, SD = 3.5) compared to the US group (mean = 7.1, SD = 3.0; *p* < 0.001). At T2, anxiety levels converged, with both groups reporting similar scores (NICT: 9.8, SD = 3.6; US: 10.5, SD = 3.8; *p* = 0.258). By T3, a significant reversal was observed: the NICT group’s anxiety had resolved nearly to baseline levels (mean = 6.1, SD = 2.9), while the US group continued to experience clinically relevant anxiety (mean = 7.8, SD = 3.3; *p* = 0.002). The LMM confirmed a significant Time-by-Group interaction for HADS-Anxiety [*F*(3, 456) = 10.8, *p* < 0.001], indicating that the trajectory of anxiety over time was fundamentally different between the two groups (Table 4).

The depression subscale revealed a pattern consistent with the hypothesis of a differential grief trajectory. Baseline depression scores were similar between groups (NICT: 4.3, SD = 2.1; US: 4.8, SD = 2.4; *p* = 0.198). At T1, an approaching but not reaching statistical significance was observed in the NICT group (mean = 6.6, SD = 3.0) compared to the US group (mean = 5.8, SD = 2.7; *p* = 0.092). At T2, both groups experienced their peak depressive symptoms, with the US group reporting slightly higher scores (mean = 9.6, SD = 3.4) than the NICT group (mean = 8.7, SD = 3.2), although this difference did not reach statistical significance (*p* = 0.105). By T3, a clear divergence emerged: the NICT group demonstrated substantial improvement (mean = 6.1, SD = 2.8), while depressive symptoms remained significantly elevated in the US group (mean = 7.7, SD = 3.1; *p* = 0.001). The LMM confirmed a significant Time-by-Group interaction for HADS-Depression [*F*(3, 456) = 6.9, *p* < 0.001], supporting the interpretation that the two groups experienced fundamentally different trajectories of depressive symptoms throughout the treatment and recovery process (Table 5).

### Qualitative findings (thematic analysis)

**Figure 4:**
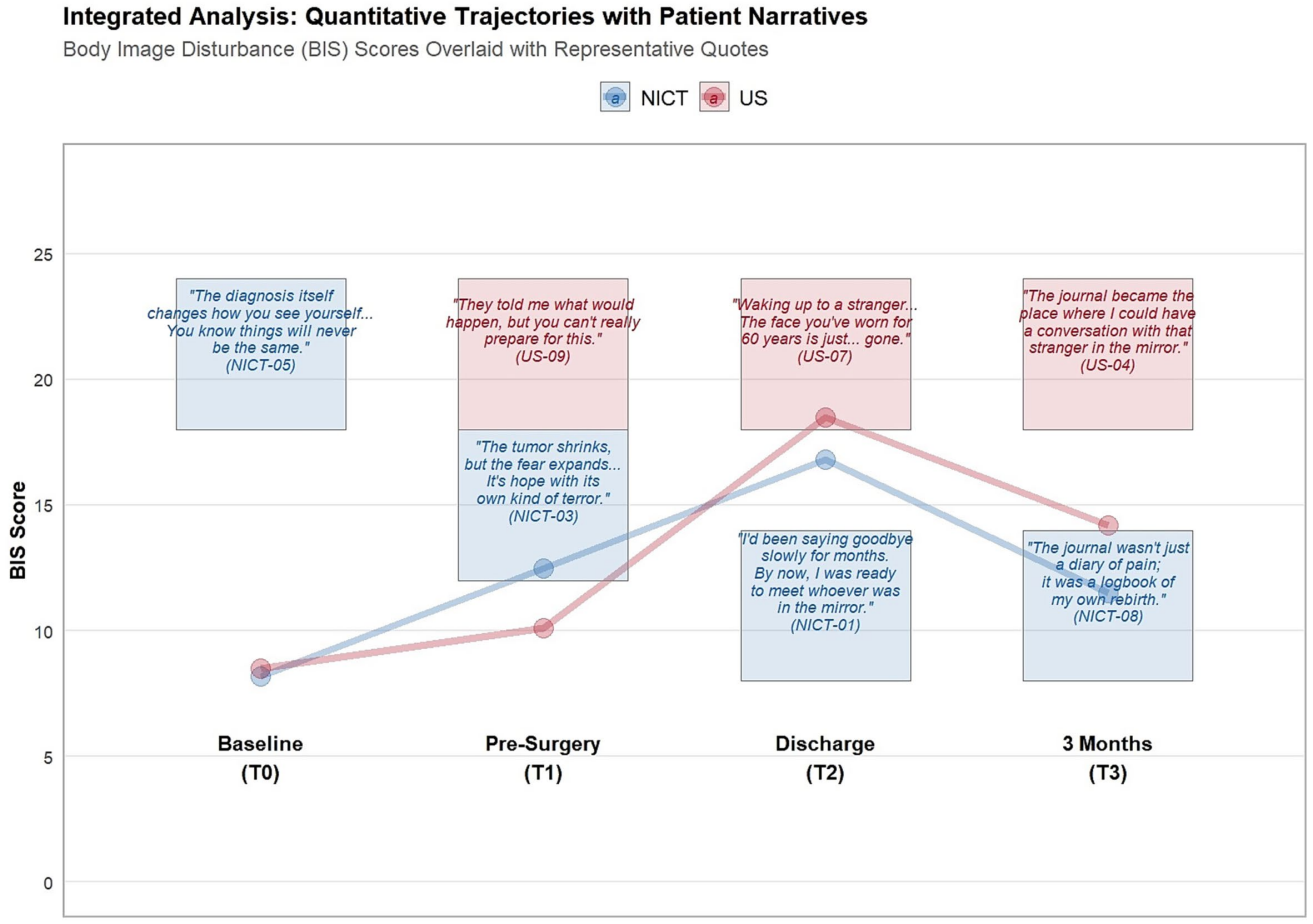
Integrated mixed-methods joint display: quantitative trajectories contextualized by patient narratives.

**Figure 5:**
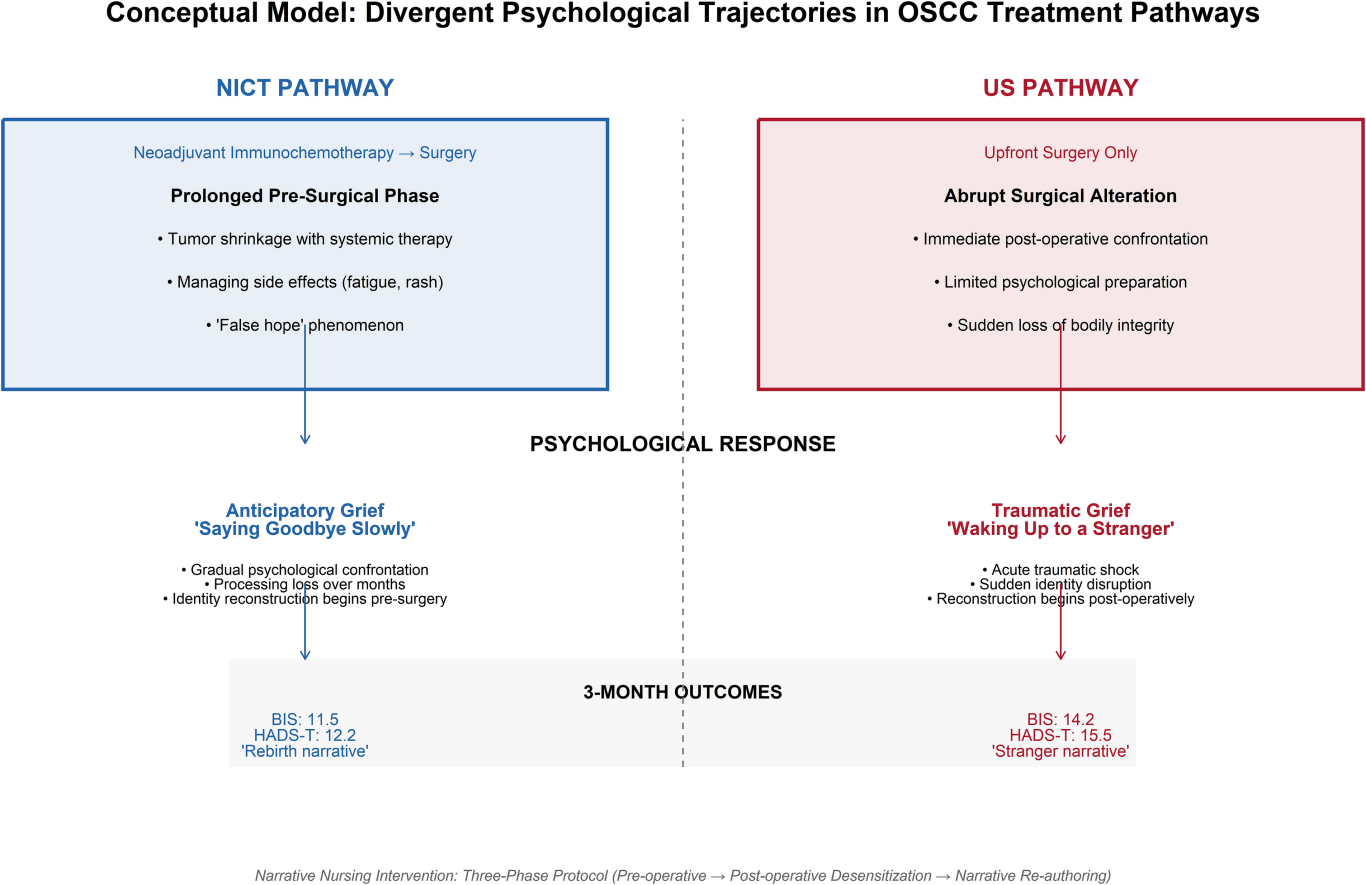
Conceptual model of divergent psychological trajectories in oral squamous cell carcinoma treatment pathways.

The qualitative component, guided by Reflexive Thematic Analysis of 15 interviews (8 NICT, 7 US), constructed four overarching themes illuminating the divergent psychological experiences of the two treatment pathways (Figures 4, 5).

#### Theme 1: the duality of waiting: “the tumor shrinks, but the fear expands” (NICT)

*For Sub-theme 1a*: The paradox of visible response.

NICT participants consistently described feeling trapped between oncological relief and emotional dread. As the tumor visibly shrank, the inevitability of major surgery became more, not less, real.

“Every time the doctor said ‘good response,’ my stomach dropped. A smaller tumor meant the surgery was still coming. It felt like being pardoned and sentenced on the same day.” (NICT-03, female, age 52).

“My family would celebrate the scan results. I would smile and then lie awake all night. The hope was real, but so was the terror. It’s a very strange kind of hope, one that comes with its own kind of terror.” (NICT-06, male, age 48).

*Sub-theme 1b*: The hidden burden of systemic toxicity.

Beyond tumor response, NICT participants endured physical side effects including fatigue, rash, oral mucositis that functioned as daily reminders of their illness. These symptoms created an “invisible suffering” that family members often failed to recognize.

“The rash on my face was manageable, but the exhaustion… no one could see that. My wife thought I was being lazy. I could not explain that my body was fighting a war it was losing, even as the tumor shrank.” (NICT-01, male, age 60).

“The mouth sores meant I could not eat with my family. I sat alone, drinking nutritional supplements, watching them laugh. That isolation was worse than the pain.” (NICT-07, female, age 45).

*Sub-theme 1c*: Anticipatory grief as slow farewell.

Crucially, the prolonged NICT window allowed participants to begin mourning their pre-illness identity before surgery. This “slow goodbye” was painful but ultimately preparatory.

“By the time I got to surgery, I’d already been processing the loss in my mind for months. I had looked at old photos. I had cried. I had told my face goodbye. When I saw myself after surgery, it wasn’t a complete shock. It was more like… meeting someone I had been expecting.” (NICT-01).

“I started writing letters to my old face. That sounds crazy, I know. But it helped. When the bandages came off, I felt sad, not destroyed. The grief had already done its work.” (NICT-08, male, age 55).

#### Theme 2: the abrupt shock of the altered self: “waking up to a stranger” (US)

*Sub-theme 2a*: Mirror avoidance and sensory dissonance.

US participants described active avoidance of their reflection and a profound sense of bodily unfamiliarity that extended beyond visual appearance.

“You see this person, and you know logically it’s you, but the reflection is a stranger… The face you have worn for 60 years is just… gone.” (US-07, male, age 62).

“I avoided mirrors for 2 weeks. My wife had to help me brush my teeth because I could not look. Even now, I turn my face away when I walk past a window.” (US-12, female, age 58).

“The first time I saw myself, I screamed. Actually screamed. The nurse thought I was in pain. I wasn’t in physical pain. I was in soul pain.” (US-03, male, age 51).

*Sub-theme 2b*: The inadequacy of preoperative counseling.

Despite standard preoperative education, US participants felt fundamentally unprepared for the sensory and emotional reality of their new appearance.

“The surgeon showed me diagrams. He explained the flap. But a drawing on paper is not the same as looking in the mirror. Nothing can prepare you for that moment.” (US-11, female, age 63).

“They said I would look different. They did not say I would not recognize myself.” (US-05, male, age 55).

*Sub-theme 2c*: Traumatic loss without preparation.

The suddenness of surgical alteration left US participants with no cognitive or emotional scaffolding, resulting in what several described as a “psychic wound” distinct from physical pain.

“It was like a sudden car crash. One day you are driving, the next you are in the hospital and everything has changed. There’s no time to brace yourself. It’s a different kind of pain, and I think it’s why I’ve struggled more to pick up the pieces.” (US-11).

“I keep dreaming of my old face. In the dream, I look normal. Then I wake up, and the first thing I do is touch my cheek. It’s still not there. I have to grieve that loss every single morning.”

(US-09, male, age 49).

#### Theme 3: divergent grief trajectories: “saying goodbye slowly” vs. “mourning a sudden death”

*Sub-theme 3a*: NICT—Anticipatory grief as psychological inoculation.

The prolonged neoadjuvant phase allowed NICT participants to distribute grief over time, reducing its acute intensity post-surgery.

“I think the neoadjuvant period was my real recovery. By the time the surgery happened, I had already accepted that my face would change. The surgery just… completed what I had already started.” (NICT-04, female, age 47).

“Looking back, the hardest month was not after surgery. It was the third week of my second cycle, when I realized I was saying goodbye to the person in my wedding photos. That was the real mourning.” (NICT-02, male, age 53).

*Sub-theme 3b*: US—Traumatic grief as unprocessed wound.

US participants described a grief that remained “stuck” or “frozen,” unable to integrate because it arrived too suddenly.

“There was no warning. One day I had my face. The next day I did not. You cannot process something that happens that fast. It just sits inside you, like a stone you cannot digest.” (US-07).

“I keep thinking: if I had known, if I had had time to prepare, maybe it would not hurt this much 3 months later.” (US-14, female, age 61).

#### Theme 4: re-authoring the self: the tools of narrative reconstruction

*Sub-theme 4a*: The journal as transitional object.

The “Survival Journal” served different purposes across groups. For NICT participants, it consolidated an identity already in formation. For US participants, it functioned as emergency scaffolding after psychological collapse.

“The journal wasn’t just a diary of pain; it was a logbook of my own rebirth. I could look back and see: on day 10 I could not look in the mirror. On day 30 I could. On day 60 I smiled.” (NICT-08).

“The journal became the place where I could have a conversation with that stranger in the mirror, until 1 day, we were not strangers anymore.” (US-04, male, age 57).

“I wrote down one ‘unique outcome’ every night—one moment where I felt strong, or hopeful, or even just not terrible. Over time, those moments started to outweigh the bad ones.” (US-11).

*Sub-theme 4b*: “Buffer” mirror therapy as graded exposure.

Participants from both groups endorsed the mirror therapy protocol, but US participants described it as more emotionally intense.

“The first time the nurse asked me to look, I cried for twenty minutes. But she stayed with me. By the third time, I could look for five seconds without crying. By the tenth time, I could see myself as… a person, not just a wound.” (US-07).

“Because I had already imagined the surgery during the neoadjuvant phase, the mirror therapy felt like confirmation, not confrontation. It was easier for me than for the US patients I met in the ward.” (NICT-05, female, age 50).

*Sub-theme 4c*: Differential utility by treatment pathway.

A key insight emerged: the same intervention may work through different mechanisms depending on treatment pathway.

“For the US patients, the journal and mirror were lifelines, they were catching them after they had already fallen. For us [NICT], they were more like training wheels—helping us walk steadily on a path we had already chosen.” (NICT-08).

## Discussion

This prospective mixed-methods cohort study provides the first empirical evidence that patients with locally advanced OSCC experience fundamentally different psychological trajectories depending on whether they undergo NICT. Despite comparable baseline levels of body image disturbance and psychological distress, the two pathways diverged markedly over time, revealing a paradoxical pattern: while NICT patients endured significantly elevated distress during the pre-surgical phase—characterized by the psychological duality of tumor shrinkage juxtaposed against anticipatory grief—they demonstrated superior recovery by three months post-surgery, with significantly lower body image disturbance and psychological distress compared to their US counterparts. The qualitative findings illuminate this quantitative reversal, suggesting that the prolonged neoadjuvant period, though psychologically taxing, may serve as an inadvertent period of gradual psychological preparation, enabling patients to begin the work of mourning their pre-illness identity before the actual surgical alteration occurs. In contrast, patients undergoing US, despite experiencing lower immediate pre-surgical distress, faced a traumatic and abrupt confrontation with their altered bodies, a shock from which their psychological recovery appeared more protracted. These findings challenge the assumption that minimizing pre-treatment distress is invariably beneficial and suggest that the timing and nature of psychological suffering—rather than its mere presence—may critically shape long-term adjustment to cancer-related disfigurement.

The clinical management of locally advanced OSCC is currently undergoing a profound paradigm shift (López et al., 2025; Sim et al., 2025). Historically, the traditional US pathway precipitates an immediate and violent confrontation with a surgically altered self, fundamentally disrupting a patient’s self-identity and frequently triggering acute body image disturbance and psychological distress. Recently, however, NICT has emerged as a vital new strategy, shifting the existing paradigm of oncologic care. Clinical trials have demonstrated that NICT can yield promising early signals of tumor response, alleviating immunosuppression, reducing tumor burden, and even allowing for response-adapted surgical approaches (Imamura et al., 2025; Zhang et al., 2025). While the oncological efficacy of NICT is increasingly supported by current evidence, this novel approach fundamentally alters the traditional treatment timeline and creates a vastly different psychological environment for the patient. Unlike the sudden trauma of US, the NICT pathway introduces a prolonged, multi-cycle pre-surgical phase characterized by a profound psychological duality. Furthermore, they are forced to endure the systemic side effects of immunochemotherapy while simultaneously navigating anticipatory anxiety and the gradual grief associated with their impending, inevitable surgical disfigurement. Despite these stark phenomenological differences between the US and NICT clinical pathways, current psychosocial care models rarely differentiate between the unique psychological trajectories of these two cohorts. The distinct psychological implications of this altered timeline remain a critical gap in the literature, underscoring an urgent need to map these divergent trajectories to inform optimally timed, pathway-specific support strategies.

The findings of this study reveal that the psychological burden of OSCC treatment is not monolithic; rather, it is fundamentally shaped by the clinical timeline. Head and neck cancer and its subsequent treatments are well-documented sources of profound body image distress, as the face and neck are central to social interaction and self-identity (Balogun et al., 2024). Our data demonstrate that patients undergoing US and NICT experience distinctly divergent psychological trajectories. For the US cohort, the immediate transition from diagnosis to radical surgical intervention results in an acute psychological trauma. These patients undergo complex reconstructions that not only alter aesthetics but can also result in significant functional impairments, such as difficulties with swallowing and speech (Bozec et al., 2020). Because the physical transformation is sudden, these patients lack the requisite time to psychologically prepare, leading to the phenomenon our qualitative data described as “waking up to a stranger.” Consequently, they exhibit sustained elevations in depressive symptoms and body image disturbance at the three-month follow-up, struggling to integrate their abruptly altered physical reality. Conversely, the NICT pathway fundamentally alters this psychological timeline. During the multi-cycle infusion phase, patients experience visible physiological changes, including early tumor shrinkage and systemic treatment effects (Elaldi et al., 2021). While this provides oncological encouragement, it simultaneously induces a state of profound psychological duality and “false hope,” as evidenced by the significant spike in anxiety and total distress prior to surgery (T1). This heightened preoperative anxiety underscores the critical necessity for early psychological screening and proactive distress management within the clinical oncology setting (Kunz et al., 2021). However, our data suggest that this prolonged period of heightened distress paradoxically serves as a vital buffer. It facilitates “anticipatory grief,” granting patients the cognitive space to slowly mourn their pre-illness identity and mentally rehearse the impending surgical changes. As a result, the NICT group demonstrated superior long-term psychological recovery and a more robust adaptation to their altered body image. The divergence in these trajectories highlights a critical clinical imperative: unchecked depression and anxiety profoundly deteriorate the overall health-related quality of life in adult cancer survivors (Zhu et al., 2024). Therefore, applying a uniform psychosocial intervention to all OSCC patients is insufficient. Support strategies must be pathway-specific. For the NICT group, early interventions incorporating mindfulness-based stress reduction and proactive cognitive coping techniques are essential to manage the intense anticipatory anxiety of the neoadjuvant phase (Schell et al., 2018). Conversely, the US group requires intensive, post-operative progressive desensitization to mitigate the acute trauma of sudden disfigurement. By utilizing tools like the “Three-Phase Narrative Reconstruction,” clinicians can effectively guide patients through their specific grief trajectories, ultimately transforming the passive endurance of surgical morbidity into the active re-authoring of the self.

The profound disruption of self-identity following the diagnosis and treatment of OSCC necessitates interventions that transcend standard medical management. A diagnosis of cancer, compounded by the physiological toll of treatments such as systemic chemotherapy and radical surgery, shatters a patient’s internal narrative, frequently precipitating an identity crisis and severe psychosocial distress (Braga Mendonça et al., 2022; Körner et al., 2016). For head and neck cancer patients, the physical manifestations of treatment are acutely visible, leading to overwhelming body image disturbance and the traumatic, isolating experience of “not recognizing oneself” (Gibson et al., 2021). Our “Three-Phase Narrative Reconstruction” protocol is theoretically grounded in narrative and acceptance-based psychotherapies, which posit that psychological healing requires patients to actively dismantle these problem-saturated stories and re-author a cohesive, empowered post-illness identity.

Phase I of our intervention focuses on proactive distress screening and visual cognitive rehearsal. The implementation of structured, tailored psychosocial screening protocols early in the clinical pathway is essential to capture the acute anticipatory anxiety and unique emotional vulnerabilities of patients, particularly those navigating the prolonged uncertainty of the NICT pathway (Brauer et al., 2022). By identifying elevated distress thresholds prior to surgical intervention, clinicians can initiate timely, targeted dialogues using validated tools like the Distress Thermometer to help patients confront the dualities of “false hope” and treatment-related fear (van der Meulen et al., 2018). This early engagement is especially critical for older demographics and those with complex unmet supportive care needs, ensuring they are not overwhelmed by the impending physical changes (McDowell et al., 2022).

Phase II introduces “Buffer” Mirror Therapy and progressive desensitization, bridging the gap between the preoperative self and the postoperative reality. Rather than avoiding the altered physical state, this phase encourages gradual, guided exposure to the surgical site. Drawing from the principles of acceptance and commitment therapy tailored for head and neck oncology, this technique reduces experiential avoidance and helps patients decouple their core self-worth from their physical disfigurement (Xu et al., 2024). By deliberately externalizing the trauma, patients learn to view their surgical scars not as symbols of loss, but as testaments to endurance, thereby mitigating the acute shock typically experienced by the US cohort.

Finally, Phase III focuses on narrative re-authoring and the reconstruction of relational dynamics. The burden of OSCC is not borne by the patient alone; it profoundly impacts the familial ecosystem, generating significant psychological distress and caregiving burden among family members (Castellanos et al., 2019). Culturally attuned care must address these systemic challenges, as the quality of family support directly influences the patient’s narrative recovery (Liang et al., 2019). Through the curation of a “Survival Journal” and guided reflective dialogues, patients and their caregivers collaboratively document “unique outcomes”—moments of resilience that contradict the dominant illness narrative. This active meaning-making process empowers patients to integrate their trauma, fundamentally transforming them from passive recipients of surgical morbidity into active authors of their renewed lives.

Comparisons with other malignancies highlight the unique psychological burden of OSCC. In stark contrast to our findings, a recent study by Pompili et al. (Pompili et al., 2026) found no significant differences in long-term anxiety or depression between NICT and upfront surgery in non-small cell lung cancer. This divergence underscores the site-specific trauma of maxillofacial surgery. Unlike internal thoracic resections, OSCC surgery inflicts inescapable facial disfigurement that fundamentally alters a patient’s social identity. Therefore, while NICT demonstrates baseline psychological tolerability in broader oncology, our data suggest that the anticipatory grief buffer afforded by NICT may be particularly valuable in head and neck cancer, where visible disfigurement is unavoidable and a protective pattern not observed in internal malignancies. For OSCC patients, the prolonged neoadjuvant phase provides an essential “anticipatory grief” buffer, granting them the crucial time to mentally prepare for severe body image alterations—a protective mechanism far less required in internal malignancies.

This study has several limitations. First, the non-randomized design limits causal inference despite propensity score matching. Second, the single-center setting in China may restrict generalizability. Third, the three-month follow-up is insufficient to assess long-term outcomes. Fourth, tumor response to neoadjuvant therapy was not examined as a potential confounder. Finally, variability in intervention delivery across nurses cannot be excluded, and also qualitative findings may not fully represent the broader cohort, as participants who agreed to interviews might have differed in psychological resilience or distress levels.

In summary, OSCC patients may experience distinct psychological trajectories depending on their treatment pathway. While NICT patients exhibited elevated pre-surgical distress, they demonstrated a more favorable psychological recovery by 3 months. Conversely, US patients experienced acute postoperative distress with a more protracted adjustment period. These findings highlight the potential value of pathway-specific psychosocial interventions rather than uniform care. The Three-Phase Narrative Reconstruction protocol may offer a useful framework for helping patients adjust to their altered post-illness identities. Future multicenter randomized trials with longer follow-up periods are warranted to confirm these observations.

## Data Availability

The original contributions presented in the study are included in the article/Supplementary material, further inquiries can be directed to the corresponding author.

## Supplementary material

SUPPLEMENTARY FIGURE S1 Pictorial representation of the mixed-methods study methodology and timeline.
