# Supplementary figures and images for: Re-authoring the altered self: the impact of a three-phase narrative nursing intervention on body image and distress in oral squamous cell carcinoma
Source: Front Psychol. 2026 May 12;17:1819611. doi: 10.3389/fpsyg.2026.1819611 (PMC13201428; doi:10.3389/fpsyg.2026.1819611)

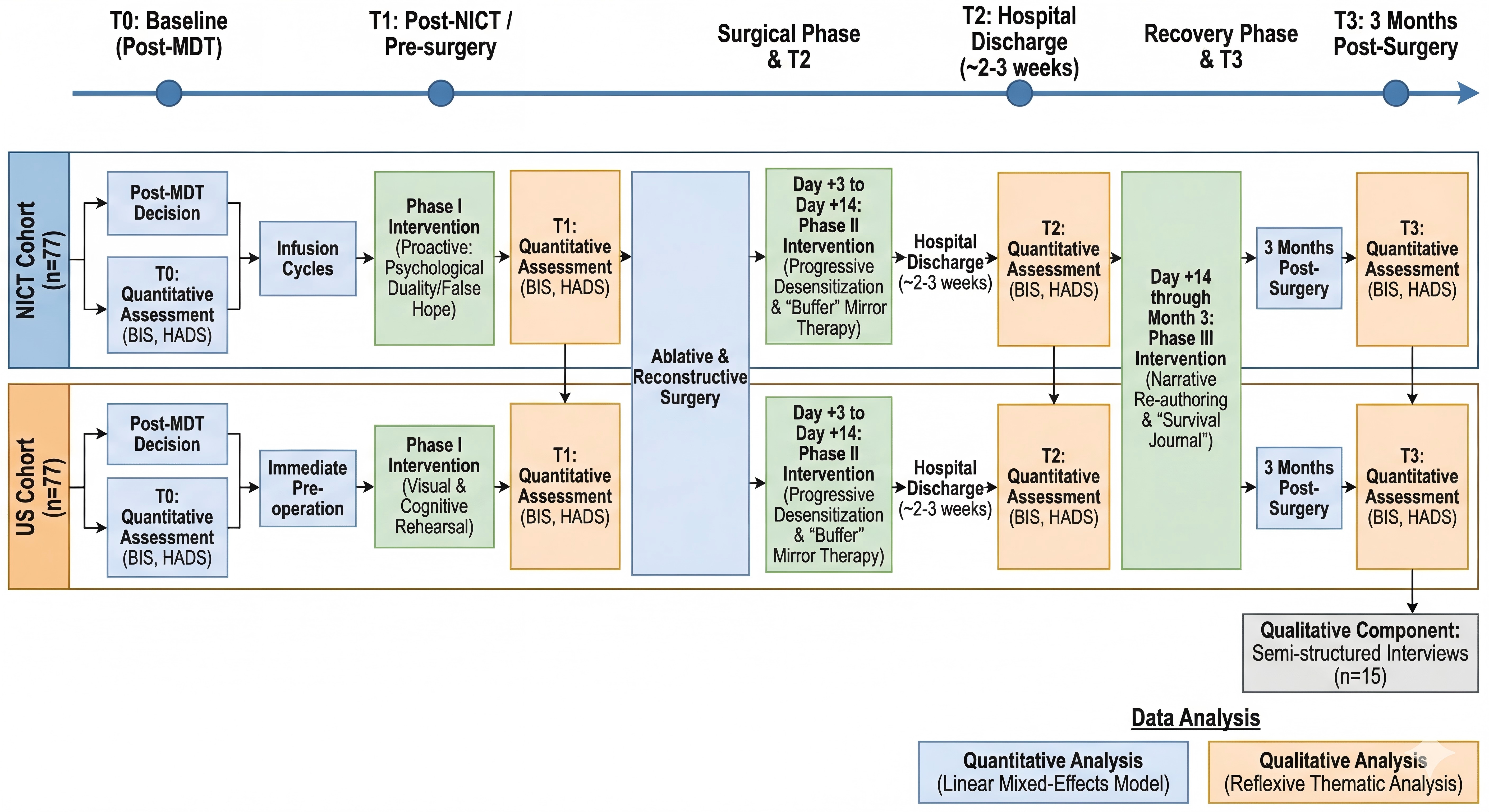

Supplement: SUPPLEMENTARY FIGURE S1 — Pictorial representation of the mixed-methods study methodology and timeline. [file Image_1.JPEG]
